# Quality and Market Potential of Olive Oils Produced in Western Santa Catarina: Physical–Chemical, Antioxidant, and Rheological Characterization

**DOI:** 10.1021/acsomega.5c03763

**Published:** 2025-07-29

**Authors:** Bruna Niedo Gerolim, João Vitor dos Santos Cruz, Matheus Henrique Roman, Idemir Citadin, Sirlei Dias Teixeira, Edimir Andrade Pereira

**Affiliations:** † Department of Chemistry, Federal Technological University of Paraná (UTFPR), Via do Conhecimento, km 01, CEP, Pato Branco, PR 85503-390, Brazil; ‡ Department of Agronomy, 651490 Federal Technological University of Paraná (UTFPR), Via do Conhecimento, km 01, CEP, Pato Branco, PR 85503-390, Brazil

## Abstract

This study detailed the quality of olive oils produced in Campo Erê, Santa Catarina—Brazil, a potential region for olive cultivation in southern Brazil. Samples were evaluated regarding physicochemical parameters (acidity, peroxide, iodine, and saponification index), fatty acid profile by gas chromatography, total phenolic compound content, and antioxidant activity (DPPH, ABTS, and FRAP methods), in addition to their rheological properties. The analyses revealed that the oils complied with current legislation, presenting a healthy lipid profile. Samples A and B1 stood out for their low atherogenicity and thrombogenicity indices and high hypocholesterolemic indices. The total phenolic compound content ranged from 245 to 295 mg gallic acid kg^–1^ and showed significant antioxidant activity. The rheological characterization indicated behavior close to Newtonian fluids, with stable viscosity under different shear rates. The experimental data fitted well with mathematical models. The overall results demonstrate the high physical–chemical quality of Campo Erê olive oils, highlighting their low acidity, favorable lipid profile, and remarkable antioxidant activity. Sample B1 showed superior performance in all parameters analyzed, suggesting great nutritional and functional potential. The rheological stability and richness in phenolic compounds reinforce the added value of these olive oils for the Brazilian market of gourmet and functional products, especially in the growing segment of healthy and controlled-origin foods.

## Graphical abstract

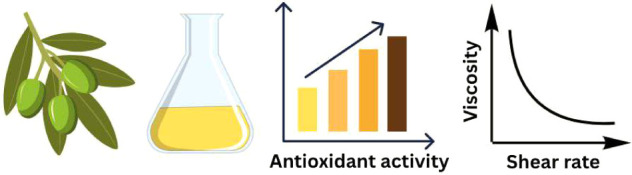

## Introduction

Olive oil is one of the world’s most traditional and valued products, widely consumed due to its sensory and nutritional properties. Its production is directly related to the cultivation of the olive tree (*Olea europaea* L.), an ancient tree that is predominant in regions with a Mediterranean climate and responsible for providing olives, the raw material for extracting the oil.^1,2^ The method of obtaining the olive oil, by exclusively mechanical or physical means, without using chemical solvents, ensures the purity and quality of the final product.^3,4^

The quality of olive oil is influenced by several factors, including the variety of olives used, the climatic conditions, the stage of ripeness, the time between harvesting and processing, and the extraction method adopted.^2,5^ Each variety of olive has distinct characteristics, which give the oil a particular sensory and chemical profile. Among the best-known cultivars are Arbequina, of Spanish origin, which produces a delicate, sweet oil with low acidity, and the Koroneiki, from Greece, distinguished by a pronounced fruity aroma.^6^ Arbequina has an easy adaptation to high-density cultivation systems and new environmental conditions, small size, precocity, high oil yield, good oil quality, and other agronomic characteristics such as branch flexibility and easy fruit abscission.^7–9^ Koroneiki produces high-quality olive oil with a distinctive fruity profile and moderate bitterness and pungency. Its aroma includes green apple, grass, and leaf notes, while the flavor is balanced yet slightly astringent, with hints of almond, fig, and bark.^10^

Soil and climate conditions, such as temperature, altitude, and rainfall patterns, also play a crucial role in the final composition of the olive oil. Countries such as Spain, Italy, Greece, and Portugal account for much of the world’s production, benefiting from an ideal climate for growing olive trees. In Brazil, olive growing has been growing, especially in the South and Southeast regions, such as Rio Grande do Sul and Serra da Mantiqueira, São Paulo, and Minas Gerais, where the climatic conditions allow the production of olive oils with qualities similar to those found in the Mediterranean.^2,11^ Recently, the western region of Santa Catarina, in Brazil, has attracted interest due to its favorable characteristics for the cultivation and production of olive oil, establishing itself as a promising new agricultural frontier.

Olive oils are classified into three main categories: extra virgin, virgin, and lampante. This classification considers free acidity, peroxide value, sensory quality, and chemical composition. Extra virgin olive oil is regarded as the highest quality, with an acidity of 0.8% or less and no sensory. In comparison, virgin olive oil can have up to 2% acidity and minor imperfections. Lampante olive oil has an acidity of over 2% and marked defects. It is intended for industrial refining and not for direct consumption.^12^

In parallel with its appreciation, the olive oil market faces significant challenges related to fraud and adulteration. High demand and high commercial value encourage illicit practices, such as mixing with lower quality vegetable oils (soybean, sunflower, or canola) or combining with refined or lampante oils.^13^ To ensure the authenticity and safety of the product, physical–chemical analyses are widely used, including acidity index, peroxides, iodine index, saponification, and fatty acid profile, often determined by gas chromatography.^13,14^

The phenolic compounds in olive oil also play a fundamental role in its quality and functionality. These bioactive substances give the oil antioxidants, anti-inflammatories, and cardioprotective properties, and influence its flavor and aroma.^4^ The main phenolics found are oleuropein, responsible for the bitter taste and antioxidant properties; hydroxytyrosol, one of the most potent natural antioxidants; tyrosol, with cardiovascular protective action; in addition to phenolic acids such as caffeic and *p*-coumaric, which contribute to the sensory profile.^15,16^ The concentration of these compounds depends on the cultivar, fruit ripeness, environmental conditions, and the extraction process.^11,17^ Among the phenolic compounds, oleuropein stands out as a bitter secoiridoid abundant in unripe olives, known for its strong antioxidant, anti-inflammatory, and antimicrobial activities. Its hydrolysis during processing leads to the formation of hydroxytyrosol, contributing to the health benefits and flavor profile of olive oil^17^

The lipid composition of olive oil, especially its fatty acid profile, is another crucial parameter for its characterization and detection of adulteration. Olive oil mainly comprises oleic acid (omega-9), which can vary between 55% and 83%, contributing to its oxidative stability and cardiovascular benefits. Other essential acids are linoleic (omega-6), linolenic (omega-3), and palmitic, whose proportions are regulated by international standards. Changes in these parameters may indicate the presence of undeclared oils, compromising the quality and authenticity of the product.^18,19^

Specific legislation has been established in several regions to guarantee the quality of olive oil and protect the consumer. In Brazil, Normative Instruction no. 1/2012 of the Ministry of Agriculture, Livestock and Food Supply^12^ defines the technical criteria for classifying olive oils, in line with the International Olive Council (IOC) guislines,^20^ Codex Alimentarius^21^ and the European Commission.^22^ The required parameters include acidity ≤ 0.8%, peroxides up to 20 mequiv O _2_ kg^–1^, iodine value between 75 and 94, saponification value between 184 and 196 mg KOH g^–1^, and defined limits for specific fatty acids.

Research has focused on the characterization of olive oils to ensure their compliance with identity and quality standards; Peralta et al. (2024) and (2025)^23,24^ studied olive oil quality, total phenolic compounds, and quantification of these compounds by liquid chromatography. Lacueva et al. (2025)^25^ studied the quality parameters of olive oils under different storage conditions. de Assis Almeida et al. (2024)^26^ characterized imported extra virgin olive oils. Xavier et al. (2025)^27^ compared olive oil quality parameters with those of soybean oil. Figueiredo et al. (2024),^28^ Abrante-Pascual et al. (2024)^29^ and Squeo et al. (2025)^30^ studied olive oils by analyzing the fatty acid profile. These advances are essential to detect adulterations and ensure the quality of olive oils available on the market.

Indicators such as the atherogenicity index (AI), the thrombogenicity index (TI), the hypocholesterolemic/hypercholesterolemic fatty acid (HH) ratio, the saturated to polyunsaturated fatty acid (SFA/PUFA) ratio, as well as the omega-6 to omega-3 fatty acid (*n* – 6/*n* – 3) ratio are used to assess the nutritional quality of the lipids present in olive oil.^31^ These indices help to assess the impact of fatty acids on health. The AI and TI indicate the risk of plaque and thrombus formation, while the HH index shows the influence on cholesterol levels, favoring the increase in HDL and the reduction in LDL. The PUFA/SFA ratio is linked to improving lipid metabolism, and the *n* – 6/*n* – 3 ratio to inflammatory balance, with lower values being desirable. Thus, these indices are helpful to assess the nutritional value of olive oil and its suitability for healthy diets.

In addition to the physicochemical and antioxidant properties, the rheological characteristics of olive oil have been gaining prominence as relevant parameters for its characterization and authentication. Rheology studies the flow and deformation behavior of fluids. Recent studies such as Charoo et al. (2023),^32^ Bayousfi et al. (2024),^33^ and Stanciu (2023),^34^ indicate that olive oil exhibits Newtonian behavior, where viscosity remains constant regardless of the applied shear rate. Rheology is closely linked to the sensory properties of olive oil, affecting attributes such as viscosity and palatability, which are crucial for consumer acceptance. Therefore, rheological characterization helps to ensure the quality and authenticity of olive oil and provides valuable insights into its functionality and sensory perception.

Given the growth of Brazilian olive and the scarcity of scientific data on regional production, especially in the Western Santa Catarina region, which has the potential to be a new frontier for olive cultivation, this study aims to evaluate the physical–chemical, antioxidant and rheological quality of olive oils produced in this region, verifying their compliance with national and international regulatory standards. Additionally, it seeks to analyze the potential for the insertion of these products in the market, contributing to the strengthening of the local production chain and the valorization of Brazilian olive oil.

## Material and Methods

### Samples

The oils were purchased from a producer in the municipality of Campo Erê, located in the state of Santa Catarina, Brazil, at an altitude of 910 m, geographic coordinates: Latitude: 26° 23′ 38″South, Longitude: 53° 4′ 40″ West,^35^ with a Cfa climate, characterized as humid subtropical with hot summers and no defined dry season,^36^ soils mostly Dystrophic Latosols, with areas of purple soil^37,38^ and an average annual rainfall of approximately 2,117 mm.^39^ Four oil samples were produced: two from single cultivars, Arbequina (A) and Koroneiki (K) and two from blends of both cultivars, labeled B1 and B2.

### Extraction of Olive Oil

The olives were harvested during the same ripening period, according to the external color of the fruit. This factor was taken into account, considering the changes in the chemical composition to which the olives are subjected at different stages of ripening.

The olive oil extraction process used by the agroindustry follows a continuous flow, with mechanical and thermal steps that aim to preserve the quality of the final product. Initially, the fruits were washed, the impurities (leaves) were separated and crushed, forming a homogeneous paste. Then, this paste was subjected to kneading (slow and controlled mixing of the mass at 28 °C) with the aim of facilitating the coalescence of the oil droplets. The separation of the oil occurs through centrifugation, using a horizontal centrifuge that allows the separation of the oil, vegetable water, and pomace. The oil was then filtered in transfer tanks, where paper filters that separate the solids were used, after which it was stored in stainless steel tanks under an inert atmosphere and controlled temperature, aiming to reduce oxidation and preserve its bioactive compounds until the time of bottling. The yield of the extraction process was calculated by the difference between the oil content in fresh fruit and the remaining oil content in the olive pomace generated in the extraction process. These measurements were performed in triplicate. The olive oil yield is calculated as eq 1:

1 $$\mathrm{Olive oil yield}\;\left(\%\right)=\frac{\mathrm{fruit oil content}-\mathrm{pomace oil content}}{\mathrm{fruit oil content}}\times 100$$

The physical–chemical analyses were performed at the Central Analysis Multiuser Laboratory at the Federal Technological University of Paraná, Pato Branco campus. The rheological analysis was performed at the Curitiba campus (Rheometry Laboratory – LabReo). The chromatography was performed in São Paulo at CBO – Laboratory Analyses Ltd.

### Acidity Index

The acidity index was calculated by titration with 0.01 mol L^–1^ potassium hydroxide from the mixture of olive oil with the alcohol/ethyl ether solution, using phenolphthalein as an indicator (eq 2).^40,41^

2 $$\mathrm{AI}=\frac{56.10\times V\times N}{m}$$

where 56.10 = gram equivalent of KOH; *V* = volume spent on titration; *N* = concentration of potassium hydroxide solution; *M* = sample weight.

### Peroxide Index

The analysis of the peroxide index occurred through titration with sodium thiosulfate 0.01 eq g L^–1^ of a mixture of olive oil with the acetic acid/chloroform solution, saturated potassium iodide, and using starch as an indicator, and blank is performed; this contains all the solutions, except the olive oil sample (eq 3).^40,41^

3 $$\mathrm{Peroxide index}=\frac{1000\times \left(V1-V2\right)\times N}{m}$$

where *V*1 = volume spent on titration; *V*2 = volume spent on blank; *N* = sodium thiosulfate concentration; *M* = sample weight.

### Saponification Index

The mixture of olive oil with an alcoholic solution of potassium hydroxide was directed to a condenser until boiling, after which it was titrated with 0.5 mol L^–1^ hydrochloric acid, a blank was performed, which contains all the solutions, except the olive oil sample (eq 4).^40,41^

4 $$\mathrm{Saponification index}=\frac{28.05\times f\times \left(B-A\right)}{m}$$

where 28.05 = gram equivalent of KOH; *f* = HCl solution factor 0.5 mol L^–1^; *B* = volume spent on blank titration; *A* = volume spent on sample titration; *m* = sample weight.

### Iodine Index

The iodine value was determined directly from the composition of unsaturated fatty acids obtained from gas chromatography analysis (eq 5).^40,41^

5 Iodine value=(% palmitoleic
acid×0.990)+(% oleic acid×0.8986)+(% linoleic acid×1.810)+(% linolenic acid×2.735)+(cis−11−eicosanoic acid×0.8175)+(% erucic acid×0.7497)

### Density

A glass pycnometer was used, whose weight was recorded while empty; water was added, and the weight was measured again. Then, each sample was placed in the pycnometer and weighed (eq 6).^40,41^

6 $$\mathrm{Density}=\frac{\mathrm{ma}-\mathrm{mv}}{\mathrm{mw}-\mathrm{mv}}$$

where ma= Mass with sample; mv = Empty mass; mw = Mass with water.

### Color Analysis

The color analysis was performed using a colorimeter; the readings were in the coordinates L*, a*, and b* and the color space L* C*, and h.^42^

### Fatty Acid Profile

The analysis was performed using a gas chromatograph equipped with a flame ionization detector (FID). The samples were subjected to transesterification for the conversion of fatty acids into methyl esters (FAMEs), according to the procedures established in the AOAC-996.06 methodology, as described by the American Oil Chemists’ Society.^40^

### Nutritional Quality Index

The nutritional quality of lipids was assessed based on the analysis of the composition of FA (fatty acids), and several indexes were used for evaluation: atherogenicity index (AI), thrombogenicity index (TI); hypocholesterolemic/hypercholesterolemic FA ratio (HH); polyunsaturated FA/saturated FA (PUFA/SSFA); and omega-6/omega-3 ratio (*n* – 6/*n* – 3).

The AI and TI indices reflect the relationship between the main saturated FAs and the main classes of unsaturated FAs. Eq 7 was used to calculate the AI, in contrast eq 8 was used to calculate the TI, where MUFA is the monounsaturated fat content, PUFA is the polyunsaturated fat content, and SFA is the saturated fat content.

7 $$\mathrm{AI}=\frac{\left(4\times C14:0\right)+C16:0}{\mathrm{MUFA}+n-6+n-3}$$

8 $$\mathrm{TI}=\frac{C14:0+C16:0+C18:0}{\left(0.5\times \mathrm{MUFA}\right)+\left(0.5\times n-6\right)+\left(3\times n-3\right)+\frac{n-3}{n-6}}$$

The HH ratio, which is associated with cholesterol metabolism, was calculated using eq 9.

9 $$\mathrm{HH}=\frac{\mathrm{C18:1}n-9+\mathrm{C18:2}n-6+\mathrm{C18:3}n-3+\mathrm{C20:5}n-3+\mathrm{C22:6}n-3}{C14:0+\mathrm{C16:0}}$$

### Rheology

The analysis was performed at the Curitiba campus (Rheometry Laboratory—LabReo). Viscosity readings were taken on a HAAKE MARS III rheometer, with coaxial cylinder geometry, the CC25 ME Ti, at controlled temperatures, ranging from 10 to 50 °C, with 10 °C increments. In each temperature range, the viscosity was determined automatically, with a shear rate variation from 0.1 to 300 s^–1^, in which two consecutive increasing and decreasing ramps were applied.

### Total Phenolic Compounds

Phenolic compounds were extracted using 80% (v/v) methanol as solvent. The sample was centrifuged at 3500 rpm, and the supernatant was carefully collected. This procedure was repeated twice more, and the extracts obtained were pooled and transferred to a 5 mL volumetric flask, completing the volume with the same solvent following the methodology described by Pizarro et al. (2013).^43^

The extracted sample was homogenized by vortexing with Folin Ciocateu reagent and distilled water to quantify total phenolic compounds. After an initial reaction period, 20% (m/v) sodium carbonate was added and left to stand for 2 h, protected from light. The absorbance was read at 765 nm using a UV–vis spectrophotometer (ESPEC-V-5000, Tecnal). Gallic acid was used for the calibration curve.^44^

### DPPH

For the analysis of the antioxidant capacity by the DPPH method, the sample was homogenized in a vortex with the 0.5 mmol L^–1^ ethanolic solution of DPPH. The mixture was left to stand for 1 h, protected from light. Then, the absorbance was read at 517 nm in a UV–vis spectrophotometer. The calibration curve was constructed using the Trolox standard.^45^

### FRAP

The FRAP reagent was prepared by mixing an aqueous solution of ferric chloride (20 mmol L^–1^), TPTZ solution (10 mmol L^–1^), and acetate buffer (0.3 mol L^–1^, pH 3.6). This FRAP reagent was added to the sample and incubated in a water bath at 37 °C for 30 min, protected from light. The reading was performed at 595 nm in the UV–vis spectrophotometer. The curve was prepared with FRAP reagent.^46^

### ABTS

The ABTS radical was prepared by the reaction between a stock solution of ABTS and potassium persulfate solution, kept at rest for 16 h in the dark. After this period, the solution was diluted with ethanol until reaching an absorbance of 0.700 nm, read at a wavelength of 734 nm. The sample was mixed with the radical, kept for 6 min, and protected from light. The absorbance was read at 734 nm in the UV–vis spectrophotometer. The calibration curve was constructed with a Trolox standard.^47^

### Statistical Treatment

The experimental data were subjected to analysis of variance (ANOVA) for comparison between groups to verify the existence of significant differences between samples. For multiple comparisons of means, the Tukey test was used (*p* ≤ 0.05). Statistical analyses were performed using Statistica software version 12.

## Results and Discussion

The average yield of olive oil obtained was 11% for Arbequina and 11.5% for Koroneiki. Similar extraction yields have been reported in the literature. In studies conducted across several municipalities in Santa Catarina, Brazil, oil yields ranged from 12.33% to 12.51% for Arbequina and 11.07% to 12.69% for Koroneiki.^48^ In Argentina, a yield of 12% for Arbequina is commonly used as a reference value.^49^ However, higher yields of 17.7% for Arbequina and 15% for Koroneiki have also been reported under specific conditions in Italy.^50^

Table 1 presents the results obtained for the physical and physicochemical analyses of the four olive oil samples (K, A, B1, and B2), including density, acidity index, peroxide index, iodine index and saponification index.

**Table 1:** Results Obtained in the Physical and Physicochemical Analyses for Samples K, A, B1 and B2^a^

Oils	Density (g cm^–3^)	Acidity index (%)	Peroxide Index (mEq kg^–1^)	Iodine value (%)	Saponification index (mg KOH g^–1^)
K	0.9124 ± 0.02^b^	0.37 ± 0.01^b^	12.92 ± 2.03^b^	78.24	188.33 ± 0.58^b^
A	0.9128 ± 0.02^b^	0.23 ± 0.01^d^	19.16 ± 0.63^a^	78.95	185.56 ± 0.05^c^
B1	0.9145 ± 0.02^a^	0.58 ± 0.01^a^	18.49 ± 1.06^a^	78.04	190.41 ± 0.03^a^
B2	0.9144 ± 0.02^a^	0.31 ± 0.01^c^	19.76 ± 1.78^a^	76.47	187.40 ± 0.26^b^
Legal standards*	0.910–0.916	≤0.8	≤20.0	75–94	184–196

a Averages followed by different letters, in the column, differ from each other by the Tukey test (*p* ≤ 0.05).

All samples met the criteria established in Normative Instruction No. 1 of January 2012,^12^ and the specifications of the *Codex Alimentarius* and the European Commission, which indicates compliance with the standards required for extra virgin olive oils.

Regarding density, the values varied from 0.9124 g cm^–3^ (B2) to 0.9145 g cm^–3^ (B1), all within the normative range. Samples B and F presented significantly higher densities (*p* ≤ 0.05), and they differed from the others, possibly related to the higher content of saturated fatty acids and higher molecular mass compounds. Xavier et al. (2025)^27^ obtained a density value of 0.91 g cm^–3^ for extra virgin olive oil and 0.92 g cm^–3^ for soybean oil. de Assis Almeida et al. (2024)^26^ obtained values of extra virgin olive oils from 0.913 to 0.922 g cm^–3^. For Andrade et al. (2017)^51^ the values obtained range from 0.901 to 0.916 g cm^–3^.

The acidity index, which assesses the degree of triglyceride hydrolysis, presented values between 0.23% (A) and 0.58% (B1), all below the maximum permitted limit of 0.8% for extra virgin olive oils. The lower the value, the better; thus, sample A stands out, with 0.23%, which suggests better conservation of the raw material and less degradation during processing. Similar acidity values were reported in the literature, ranging from 0.2 to 0.6% in olive oils analyzed by Alves de Carvalho et al, (2020),^52^ 0.3 to 0.4% by Mansouri et al. (2019),^53^ 0.24 to 0.3% by Peralta et al. (2024)^23^ and 1.1 to 3.8% by Lacueva et al. (2025),^25^ and 0.3 by Ghorbel et al. (2025),^54^ confirming the adequacy of the results observed in this study to the standards described in the literature. All samples differ according to the Tukey test (*p* ≤ 0.05).

The peroxide index values, which indicate the primary oxidation of lipids, ranged from 12.92 mEq kg^–1^ (K) to 19.76 mEq kg^–1^ (B2). All samples were below the limit of 20 mEq kg^–1^, showing that the oils maintain good oxidative stability. Sample B2 has the best value (12.92 mEq kg^–1^). The values obtained are in agreement with the literature, 5 to 19 mEq kg^–1^ by Alves de Carvalho et al. (2020),^52^ 7 to 9 mEq kg^–1^ by Mansouri et al. (2019),^53^ 8 to 12 mEq kg^–1^ by Theodosi et al. (2021)^55^ and 11 to 39 11 to 39 mEq kg^–1^ by de Assis Almeida et al. (2024).^26^ For Xavier et al. (2025)^27^ the olive oil obtained a value of 11.04 mEq kg^–1^ and soybean oil 5.52 mEq kg^–1^. For Ghorbel et al. (2025)^54^ the value for extra virgin olive oil was 7.82 mEq kg^–1^ and for virgin olive oil 9.70 mEq kg^–1^.

The iodine index, related to the degree of unsaturation of fatty acids, showed slight variation between samples from 76.4 7% (B2) to 78.940% (B1), remaining within the normative range (75 to 94%). This reinforces the characteristic of the predominance of monounsaturated fatty acids in the olive oils evaluated. Data reported in the literature with values ranging from 77 to 91% (Barros, 2019),^56^ from 75 to 125% (Almeida et al., 2024)^26^ and from 37 to 94% (Lacueva et al., 2025).^25^

For the saponification index, which reflects the length of the fatty acid chains, varied between 185.56 mg KOH g^–1^ (A) and 190,41 mg KOH g^–1^ (B1), all the normative range (184 to 196 mg KOH g^–1^). The higher value observed in sample B1 suggests the relatively greater presence of shorter-chain fatty acids, which may positively influence the stability and texture of the olive oil. de Assis Almeida et al. (2024)^26^ obtained values ranging from 134 to 225 mg KOH g^–1^ and Jesus (2025)^57^ obtained values ranging from 173 to 177 mg KOH g^–1^.

These results demonstrate that all samples meet the regulatory requirements for extra virgin olive oils. However, some parameters have significant differences, which may reflect variations in the cultivars used, processing conditions, or storage time.

Table 2 shows the average values obtained for the colorimetric analysis of the parameters L* (luminosity), a* (green-red component), b* (blue-yellow component), C* (chroma or saturation), and h (hue or angle), together with the standard deviation for each parameter.

**Table 2:** Results for Colorimetric Analysis^a^

	L*	a*	b*	C*	h
K	32.61 ± 0.03^c^	-0.90 ± 0.011^b^	21.49 ± 0.0061^d^	21.5 ± 0.062^d^	92.38 ± 0.025^c^
A	37.89 ± 0.05^b^	-2.42 ± 0.025^c^	31.19 ± 0.1^b^	31.28 ± 0.095^b^	94.42 ± 0.085^b^
B1	32.74 ± 0.02^d^	-0.76 ± 0.035^a^	22.53 ± 0.047^c^	22.52 ± 0.058^c^	91.87 ± 0.488^d^
B2	40.81 ± 0.051^a^	-4.29 ± 0.02^d^	32.19 ± 0.061^a^	32.47 ± 0.067^a^	97.6 ± 0.023^a^

a Averages followed by different letters, in the column, differ from each other by the Tukey test (*p* ≤ 0.05).

Luminosity values (L*) obtained varied significantly between samples, from 32.61 (K) to 40.81 (B2). Sample B2 presented the highest luminosity (*p* < 0.05), indicating a lighter oil, possibly related to the lower content of pigments such as chlorophylls and pheophytins. In contrast, samples K and B1 presented lower luminosity, with darker and more intense coloration. Jesus (2025)^57^ obtained luminosity values ranging from 4.43 to 6.93. The values obtained by Cairone et al. (2021)^58^ demonstrated results between 36 and 56.

The parameter a* ranged from −4.29 (B2) to −0.76 (B1), indicating that all samples presented green tones. Sample B2 stood out with the most negative value, which suggests a greater presence of green pigments, possibly originating from less ripe fruits. Jesus (2025)^57^ obtained values ranging from 0.43 to 0.63. Cairone et al. (2021)^58^ reported values that are very close to 0.

The b* parameter, which expresses the intensity of the yellow color, varied between 21.49 (K) and 32.19 (B2), the latter being significantly higher (*p* < 0.05). This indicates a more yellow coloration in sample F, which can be attributed to the higher concentration of carotenoids. Jesus (2025)^57^ obtained luminosity values ranging from 3.03 to 4.10. Cairone et al. (2021)^58^ ranged from 31 to 52.

Saturation, which represents color purity, ranged from 21.50 (K) to 32.47 (B2). Sample B2 showed the highest saturation, revealing a purer color, a desirable fresher olive oils with good visual quality. Sicari et al. (2021)^59^ demonstrated a variation from 23 to 34.

The hue varied between 91.87 (B1) and 97.6 (B2), indicating that all samples presented hues in the yellow range, typical of extra virgin olive oils. These findings are consistent with studies that associate the color of olive oil with the ripening stage of the olives, as well as with the amount of chlorophyll and carotenoids present in the olive fruit.^60^

In general, the data obtained agree with previous studies that associate variations in olive oil color with the composition of natural pigments, such as chlorophylls and carotenoids, and the degree of ripeness of the fruit. Sample B2, because it presents greater brightness, saturation and intensity of yellow, suggests origin from olives harvested at an earlier stage, with a high pigment content, which can positively impact the product’s oxidative stability and sensory appeal.

The fatty acid profile is shown in Table 3, data are expressed as a percentage.

**Table 3:** Fatty Acid Profile in Olive Oil Samples

Fatty acids	K	A	B1	B2
Myristic (C14:0)	0.01	0.01	0.01	0.01
Palmitic (C16:0)	12.77	16.11	13.46	14.81
Palmitoleic (C16:1n7)	0.98	2.06	1.19	1.16
Margaric (C17:0)	0.04	0.08	0.05	0.06
Stearic (C18:0)	2.21	1.86	2.13	2.27
Oleic (C18:1n9c)	76.64	69.92	74.86	71.1
Linoleic LA (C18:2n6c)	4.47	7.61	5.13	6.15
Alpha Linoleic LNA (C18:3n3)	0.65	0.56	0.62	0.65
Arachidic (C20:0)	0.45	0.42	0.45	0.47
cis-11-Eicosenoic (C20:1n9)	0.38	0.37	0.38	0.37
Behenic (C22:0)	0.15	0.13	0.15	0.15
Lignoceric (C24:0)	0.08	0.08	0.08	0.09
Monounsaturated fat	78.19	72.48	76.59	72.79
Polyunsaturated fat	5.14	8.18	5.76	6.81
Unsaturated fats	83.32	80.66	82.35	79.6
Saturated fats	16.83	19.64	17.42	19.01
Trans fats	0.19	0.13	0.16	0.16
Omega 3	0.65	0.56	0.62	0.65
Omega 6	4.47	7.61	5.13	6.15
Omega 9	77.22	70.43	75.4	71.63
Ethereal Extract	100.15	100.3	99.77	98.61

The data demonstrate that all samples comply with the limits established by Brazilian legislation,^12^ the Codex Alimentarius^21^ and the European Commission,^22^ with no evidence of adulteration or significant deviations from the typical composition of extra virgin olive oils.

Sample K stood out for presenting a higher concentration of oleic fatty acid (76.64%), in addition to the highest content of monounsaturated fats (MUFA, 78.19%) and unsaturated fats (83.32%). These values are associated with better oxidative stability and greater benefits to cardiovascular health due to the proven action of MUFAs in reducing LDL cholesterol and preventing chronic diseases.^61^ Furthermore, this sample had a lower content of saturated fats (SFA, 16.83%), reinforcing its superiority from a nutritional point of view.

On the other hand, sample A presented the highest content of palmitic acid (C16:0, 16.11%) and PUFA (8.18%), which may indicate lower oxidative stability since polyunsaturated fatty acids are more susceptible to degradation. Sample B2 presented a similar profile, emphasizing the second-highest value of PUFA (6.81%) and a lower value of MUFA (72.79%). Sample B1 presented a balance between MUFA (76.59%), PUFA (5.76%), and an intermediate content of oleic acid (74.86%), positioning itself as an option with good nutritional performance and stability.

The values found in the literature and those observed in this work are in agreement, Mansouri et al. (2019)^53^ carried out studies on the cultivars Arbequina, Koroneiki and Arbosana, reporting value ranges similar to those observed in this study. C16:0 ranges from 13 to 16%, C18:1 ranges from 67 to 76%, unsaturated fats from 70 to 78% and saturated fats from 15 to 19%. On the other hand, Alves de Carvalho et al. (2020)^52^ analyzed the Brazilian Arbequina and Koroneiki variations in the Southeast and South regions and Spanish Arbequina, C16:0 varies from 8 to 17% and has a higher value for the Arbequina cultivar from the South region of Brazil. C18:1 varies from 66 to 86%, with higher values for the Koroneiki cultivar. Squeo et al. (2025)^30^ obtained values ranging from 10 to 19% for C16:0 and oleic acid varied from 61 to 81%. Abrante-Pascual et al. (2024)^29^ obtained oleic acid values from 55 to 83%, and 7.5 to 20% for C16:0. Figueiredo et al. (2024)^28^ obtained values for the Arbequina cultivar of oleic acid 71%, C16:0 of 16%, MUFA 73%, PUFA 8% and SFA 18%. For Koroneiki, the oleic acid was 73%, C16:0 of 14%, MUFA 74%, PUFA8% and SFA 17%. Ghorbel et al. (2025)^54^ presented oleic acid values for virgin olive oil of 62%, C16:0 of 16%, SFA of 16%, PUFA of 16% and MUFA of 64% and for extra virgin olive oil, the oleic acid was 60%, C16:0 of 17%, SFA of 21%, PUFA of 16% and MUFA of 62%.

Thus, the data obtained in this study confirm the sample’s authenticity and highlight sample K as nutritionally better, due to its high oleic acid content, lower saturated fat content, and balanced lipid profile.

### Nutritional Properties

Nutritional quality indices of the lipids in the olive oil samples are shown in Table 4.

**Table 4:** Nutritional Quality Indices for Samples K, A, B1 and B2

Sample	PUFA/SFA	*n* – 6/*n* – 3	AI	TI	HH
K	0.31	6.88	0.15	0.35	6.38
A	0.42	13.59	0.20	0.43	4.84
B1	0.33	8.27	0.16	0.36	5.97
B2	0.36	9.46	0.19	0.41	5.24

The nutritional quality of the analyzed olive oil samples was evaluated using classical lipid quality indices, namely the PUFA/SFA ratio (polyunsaturated/saturated fatty acids), the *n* – 6/*n* – 3 ratio, the atherogenicity index (AI), the thrombogenicity index (TI) and the hypo/hypercholesterolemic ratio (HH). These parameters provide essential insight into the potential health impacts associated with the fatty acid composition of these olive oils.

The PUFA/SFA ratio ranged from 0.31 (sample K) to 0.42 (sample A). Although these values are below those of oils rich in polyunsaturated fats, they are consistent with olive oil’s predominant profile of monounsaturated fatty acid characteristic. Higher values of this ratio are associated with better lipid metabolism and cardiovascular protection; in this sense, sample A presented the most favorable value.

The *n* – 6/*n* – 3 ratio ranged from 6.88 (sample K) to 13.59 (sample A). Lower values are nutritionally better due to the greater presence of omega-3, which has anti-inflammatory effects. Sample K stood out with the best balance between omega-6 and omega-3 fatty acids, which may contribute to protective effects against chronic inflammatory diseases. In contrast, sample A presented the highest ratio, which suggesting a less balanced lipid profile.

Regarding the indices associated with cardiovascular risk, all samples presented low values of AI (0.15 to 0.20) and TI (0.35 to 0.43), which indicates a lipid profile with low atherogenic and thrombogenic potential, consistent with the literature that associates olive oil consumption with cardioprotective effects.

The HH index, which evaluates the proportion between hypo and hypercholesterolemic fatty acids, ranged from 4.84 (sample A) to 6.38 (sample K). Sample K demonstrated the best hypocholesterolemic potential, indicating a higher proportion of fatty acids that contribute reducing of serum cholesterol levels, mainly due to the high oleic acid content.

The data obtained suggest that all samples have desirable nutritional characteristics, emphasizing sample K, which presented the most balanced set of indices, combining low atherogenicity and thrombogenicity with a high hypocholesterolemic capacity. These results reinforce the nutritional value of olive oil as an essential component of a balanced diet and prevention of cardiovascular and metabolic diseases.

The comparison of nutritional indices with data from Tilami et al. (2022)^62^ and Figueiredo et al. (2024)^28,62^ revealed similarities between lipid profiles, with PUFA/SFA ratios and AI, TI, and HH values close between the studies. The *n* – 6/*n* – 3 ratio also presented values whose ideal balance favors lower values. The results confirm that local olive oils have nutritional quality comparable to other producing regions, with emphasizing on sample K, which presented the best functional indices.^28,62^

### Phenolic Compounds and Antioxidant Activity

Table 5 presents the results of the analyses of total phenolic compounds and antioxidant capacity of the olive oil samples, obtained using the ABTS, DPPH, and FRAP methods, together with the standard curve corresponding to each method.

**Table 5:** Results Obtained for Total Phenolic Compounds, ABTS, DPPH, and FRAP^a^

	Total phenolics (mg gallic acid kg ^–1^)	ABTS (μmol Trolox kg ^–1^)	DPPH (μmol Trolox kg ^–1^)	FRAP (μmol Ferrous Sulfate kg ^–1^)
K	253.30 ± 1.28^b^	1260.09 ± 5.68^b^	601.26 ± 1.79^b^	2359.39 ± 8.12^b^
A	256.48 ± 1.12^b^	1326.60 ± 1.89^a^	607.78 ± 1.79^b^	2464.51 ± 8.11^a^
B1	295.52 ± 1.72^a^	1333.59 ± 3.30^a^	614.13 ± 1.72^a^	2500.73 ± 8.17^a^
B2	245.26 ± 1.95^c^	933.76 ± 1.89^c^	547.16 ± 3.58^c^	2252.09 ± 2.43^c^
Standard curve	*Y* = 0.0041*x* + 0.0552	*Y* = −0.0003*x* + 0.7105	*Y* = −0.0032*x* + 0.5123	*Y* = 0.0007*x* – 0.0581
	*R*^2^ = 0.9905	*R*^2^ = 0.9963	*R*^2^ = 0.9969	*R*^2^ = 0.9939

a Averages followed by different letters, in the column, differ from each other by the Tukey test (*p* ≤ 0.05).

Among the samples analyzed, sample B1 presented the highest levels of total phenolic compounds (295.52 mg gallic acid kg^–1^), followed by samples A (256.48 mg kg^–1^) and K (253.30 mg kg^–1^), which did not differ from each other and B2 (245.26 mg kg^–1^). These results indicate a higher concentration of bioactive compounds with antioxidant action in sample B1, which may be associated with the degree of ripeness of the olives, the variety used, or processing and storage conditions.

Antioxidant activity results obtained by the ABTS, DPPH, and FRAP methods confirm the profile observed in total phenolics. Sample B 1 also presented the best results in the DPPH (614.13 μmol Trolox kg^–1^) and ABTS (1333.59 μmol Trolox kg^–1^) and FRAP (2500.73 μmol Fe^2+^ kg^–1^), with statistically significant differences about the others (*p* < 0.05), except in comparison with sample A, with which it did not differ significantly in the ABTS and FRAP methods. Sample B2 presented the lowest values in all tests, standing out as the one with the lowest antioxidant potential.

The correlations between the levels of compounds and the antioxidant activity observed reinforce the contribution of these bioactives to the oxidative stability of olive oils. Phenolic compounds are known to act as free radical scavengers and to directly contribute to olive oil’s sensory and functional quality.

The results obtained in this study are by literature. A study by Santos et al. (2021)^63^ obtained values of phenolic compounds ranging from 162 to 218 mg gallic acid kg^–1^. For Peralta et al. (2025),^24^ a value of 395 mg gallic acid kg^–1^ was obtained. Barros (2019)^56^ the Koroneiki cultivar obtained a value of 217 mg of gallic acid per kg^–1^ and Arbequina 295 mg of gallic acid per kg^–1^, DPPH ranges from 8.5 to 10.7 mmol Trolox kg^–1^, FRAP 57 to 89 mmol Trolox kg^–1^ and ABTS 25 to 29 mmol Trolox kg^–1^. For Habibi et al. (2021)^64^ values of ABTS 218 μmol Trolox kg^–1^, DPPH 592 μmol Trolox kg^–1^ and FRAP 1124 μmol Trolox kg^–1^ were found. For Ghorbel et al. (2025)^54^ the values obtained for total phenolic compounds for extra virgin olive oil are 306 mg of gallic acid per kg^–1^ and virgin olive oil 208 mg of gallic acid per kg^–1^. Revelou et al. (2024)^65^ obtained values for total phenolic compounds, ABTS, and FRAP ranging from 117 to 249 mg of gallic acid per kg^–1^, from 199 to 205 mg Trolox per kg^–1^, and 1354 to 1402 mg Fe^2+^ kg^–1^, respectively.

These findings indicate that, besides being within the expected parameters for extra virgin olive oils, the samples analyzed, especially sample B, at present an excellent bioactive profile, suggesting greater potential for oxidative conservation and possible health benefits.

### Rheology

Figure 1 shows the graphs of shear stress as a function of shear rate for samples K, A, B1, and B2, evaluated at temperatures of 10 and 50 °C for the up and down ramp.

**Figure 1:**
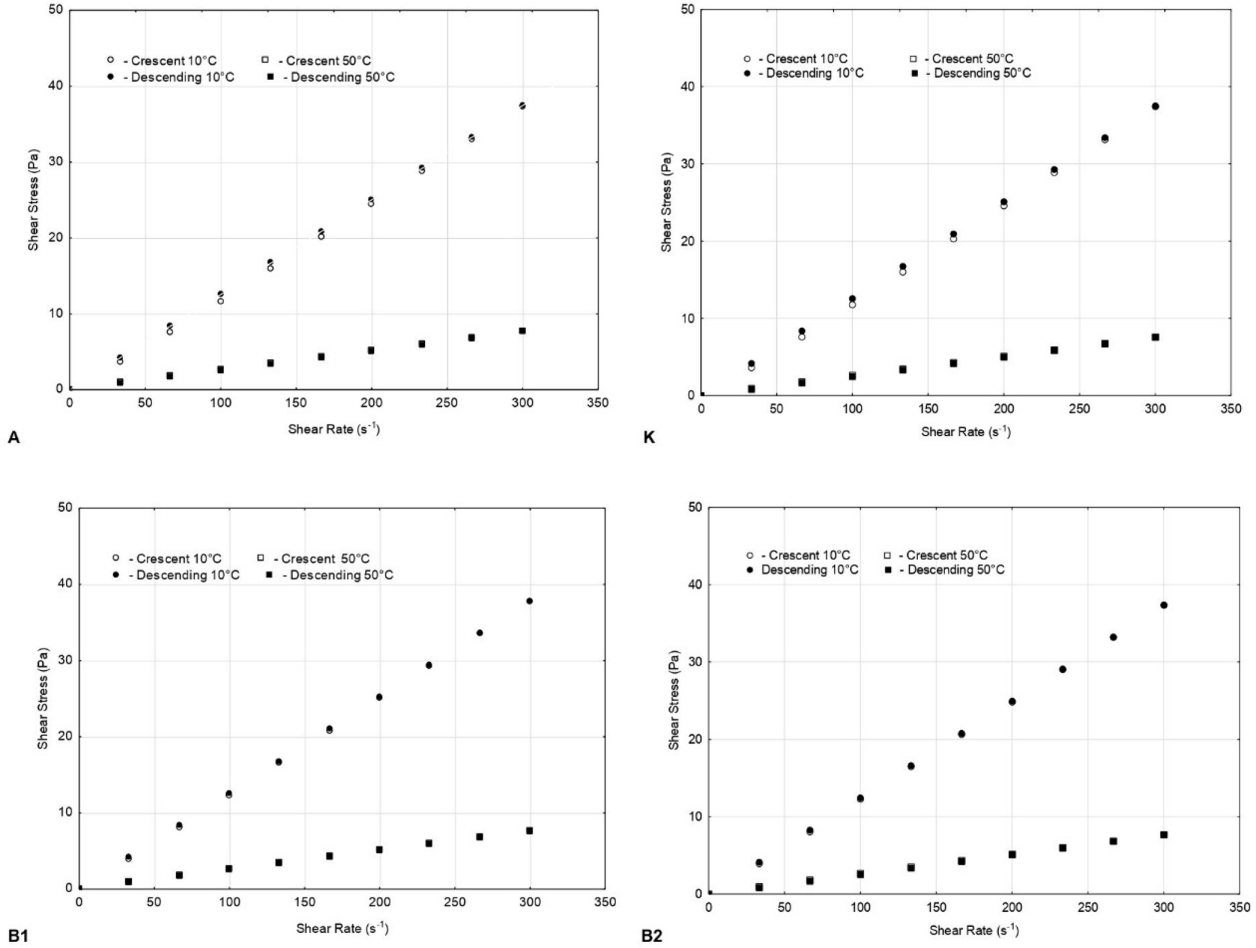
Shear stress vs shear rate plot of samples A, K, B1, and B2.

A linear response between stress and shear rate is observed, which indicates that the analyzed samples exhibit Newtonian fluid behavior, with overlap between increasing and decreasing curves. This absence of hysteresis confirms that there is no significant reversible structuring during flow, i.e., no thixotropic behavior was identified.^66^

In this work, the sample curves demonstrated stable behavior without significant changes related to the time of shear application, reinforcing the conclusion that there is no relevant thixotropic effect in the experimental conditions evaluated. Additional tests were also conducted at intermediate temperatures of 30 °C and 40 °C, which showed similar rheological behavior and thus are not displayed.

Furthermore, the linearity observed at both 10 and 50 °C suggests that the rheological properties of these samples remain stable in different temperature ranges, as in the studies by Charoo et al. (2023) and Bayousfi et al. (2024),^32,33^ which may be a favorable indication for their technological application, especially in formulations where viscosity control is critical.

Two empirical models were used, namely the Power Law (PL) and the Herschel–Bulkley (HB), for temperatures of 10, 20, 30, 40, and 50 °C. Table 6 contains the parameters for each model of the 4 samples, these being consistency index (*K*), initial stress (*T*_0_), flow behavior index (η), and *R* ^2^.

**Table 6:** Results for the Parameters of the Empirical Models LP and HB

		LP model			HB model			
Sample	Temperature (°C)	*K*	η	*R* ^2^	*T*_0_	*K*	η	*R* ^2^
A	10	0.11	1.02	0.99999	–0.05	0.12	1.01	0.99999
	20	0.10	0.97	0.99998	0.03	0.09	0.97	0.99999
	30	0.07	0.95	0.99994	0.04	0.07	0.96	0.99995
	40	0.05	0.96	0.99995	0.03	0.04	0.96	0.99996
	50	0.03	0.96	0.99996	0.02	0.03	0.97	0.99997
K	10	0.09	1.05	0.99996	–0.08	0.96	1.05	0.99997
	20	0.07	1.02	1,00000	–0.01	0.07	1.02	1,00000
	30	0.07	0.95	0.99994	0.04	0.06	0.96	0.99995
	40	0.04	0.96	0.99996	0.02	0.04	0.97	0.99996
	50	0.03	0.97	0.99996	0.02	0.03	0.97	0.99997
B1	10	0.12	1.00	0.99999	–0.01	0.12	1.00	0.99999
	20	0.08	1.01	0.99999	0.00	0.08	1.01	0.99999
	30	0.07	0.95	0.99994	0.04	0.07	0.96	0.99995
	40	0.04	0.96	0.99996	0.02	0.04	0.96	0.99996
	50	0.03	0.96	0.99996	0.02	0.03	0.97	0.99997
B2	10	0.11	1.02	0.99998	–0.01	0.11	1.02	0.99999
	20	0.07	1.02	1,00000	0.00	0.07	1.02	1,00000
	30	0.07	0.95	0.99994	0.04	0.07	0.95	0.99995
	40	0.05	0.96	0.99995	0.02	0.04	0.96	0.99996
	50	0.03	0.96	0.99996	0.02	0.03	0.96	0.99996

The index that describes the resistance to the flow of a fluid is the consistency index (*K*). In the data obtained for the four samples analyzed (A, K, B1, and B2), it is observed that the *K* index gradually decreases with increasing temperature in both models (LP and HB). This behavior is common in viscoelastic materials since, with increasing temperature, there is greater mobility of the molecules, reducing the resistance to movement and, consequently, the consistency of the material.

The flow behavior index (η) for all samples presents values close to 1. This result indicates that, under the conditions studied, the materials behave as Newtonian fluids, especially at lower temperatures (10 and 20 °C). At higher temperatures, the η values tend to reduce slightly, suggesting a slight pseudoplastic behavior.

For the Herschel–Bulkley (HB) model, there is an initial stress (*T*_0_), which represents the resistance to the beginning of the material flow. Generally, the values of *T*_0_ are close to zero or negative, reinforcing the Newtonian behavior of the samples. In addition, a tendency for *T*_0_ to decrease with increasing temperature is observed, especially for samples A and K, which indicates that the material becomes more fluid under these thermal conditions, a common phenomenon in food systems.

The *R* ^2^ value was equal to one or very close to it for both empirical models applied (LP and HB) in all samples and temperature ranges. This result reinforces the high quality of the adjustment of the experimental data to the mathematical models considered, indicating that both models are adequate to describe the rheological behavior of the evaluated samples.

Figure 2 shows between viscosity and shear rate relationship for different olive oil samples (A, K, B1, and B2) at temperatures ranging from 10 to 50 °C.

**Figure 2:**
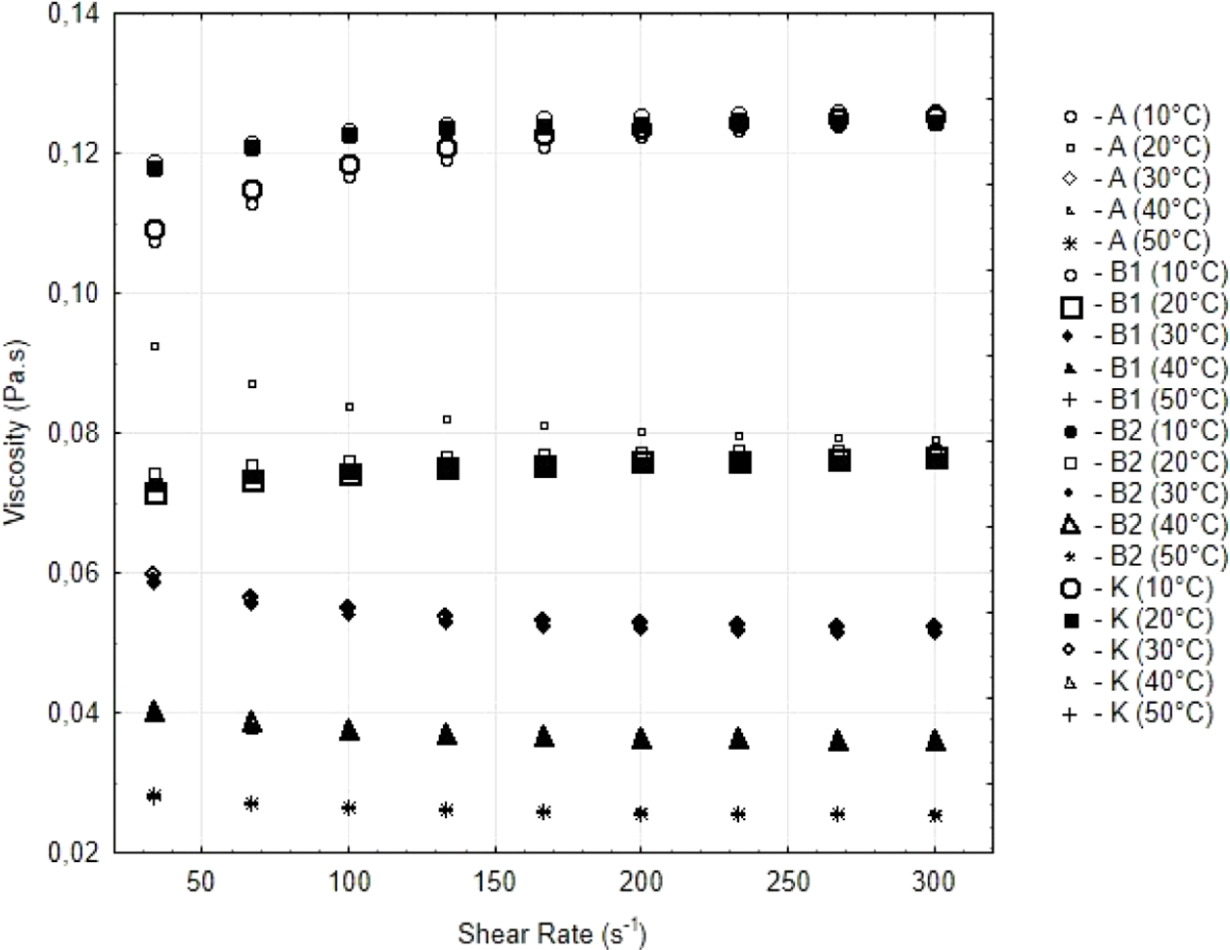
Viscosity graph by shear rate containing all samples at of 10 to 50 °C temperature.

In general, it is observed that, for all samples and conditions evaluated, the viscosity remains practically constant throughout the variation of the shear rate, with slightly inclined and stable curves, which suggests a behavior close to that of a Newtonian fluid, as observed for Charoo et al. (2023).^32^ Although there is a slight pseudoplastic tendency (typical of non-Newtonian fluids), the variation in viscosity as a function of the shear rate is not highly significant, indicating that the analyzed oils approach Newtonian behavior under the established experimental conditions.

This pattern observed in the graphs is corroborated by the results of the mathematical modeling with the Herschel–Bulkley (HB) model, in which the parameter n (fluid behavior index) presented values very close to 1 for all samples. Thus, the values of *n* ≈ 1 reinforce the visual interpretation and confirm the adequacy of the mathematical adjustment (*R* ^2^ > 0.999), demonstrating the coherence between the empirical data and the rheological model.

Temperature also significantly influenced viscosity: at lower temperatures, viscosity increased, attributed to lower molecular mobility and greater intermolecular interaction. As the temperature increased, viscosity progressively decreased, a behavior typical of vegetable oils. The differences observed between the samples, with sample A presenting the highest viscosity values and sample K the lowest, indicate variations in composition, possibly related to the fatty acid profile, phenolic compound content, or presence of minor components.

Therefore, the rheological analysis revealed that, despite the complexity of the lipid matrix of olive oils, they behave in a quasi-Newtonian manner within the temperature and shear rate ranges tested. This characteristic has relevant practical implications, especially for industrial and gastronomic applications, where predictability of flow behavior is desirable in processes such as emulsification, frying, and storage.

As observed in Table 7, the samples present activation energies ranging from 28.83 to 29.19 kJ mol^–1^; those with the highest energy are F and K, and those with the lowest energy are B1 and A.

**Table 7:** Values for Activation Energy and the Fitting Parameter

Sample	η_0_ (Pa s)	Ea (kJ mol^–1^)	R^2^
A	5.29 × 10^–7^	29.07	0.9997
K	5.67 × 10^–7^	28.83	0.9989
B1	4.95 × 10^–7^	29.19	0.9982
B2	5.53 × 10^–7^	28.94	0.9992

In a study by Gila et al. (2015),^67^ Ea values for virgin olive oils were observed to be between 30.65 and 33.63 kJ mol^–1^; for Ashrafi (2012),^68^ extra virgin olive oil obtained a value of 29.20 kJ mol^–1^ and for Marques (2015),^69^ the values ranged from 24.40 to 26.00 kJ mol^–1^.

The coefficient of determination (*R* ^2^) values for all samples are above 0.99, indicating that the fit of the Arrhenius equation to the analyzed data was excellent, thus demonstrating behavior for Newtonian fluids.

## Conclusion

Based on the integrated assessment of nutritional, functional, antioxidant, and physicochemical parameters, sample B1 stood out as the most promising among the olive oils analyzed. This sample presented the highest content of phenolic compounds (295.52 mg gallic acid kg^–1^) and the best results in the antioxidant capacity tests (DPPH = 614.13 μmol Trolox kg^–1^; FRAP = 2500.73 μmol ferrous sulfate kg^–1^), which suggests high protection against lipid oxidation.

From a nutritional point of view, sample B1 presented balanced indices, with a low atherogenicity value (AI = 0.16) and low thrombogenicity index (TI = 0.36). and high hypocholesterolemic index (HH = 5.97). In addition, its fatty acid profile was marked by a high proportion of oleic acid (74.86%) and adequate proportions of polyunsaturated fatty acids. Although the acidity index (0.58%) was the highest among the samples, the value is within the limits permitted by current legislation for extra virgin olive oils. These results show that sample B1 combines high nutritional and functional quality, being indicated as the most suitable for consumption and with the greatest potential for commercial appreciation.

The results obtained show that the olive oils produced in Campo Erê - SC have high physical and chemical quality, standing out for their low acidity levels, lipid profile compatible with standards of excellence, and notable antioxidant activity.

## Abbrevations

PUFA: polyunsaturated fatty acids
SFA: saturated fatty acids
MUFA: monounsaturated fatty acids
DPPH: 2,2-diphenyl-1-picrylhydrazyl
FRAP: ferric reducing antioxidant power
ABTS: 2,2′-azinobis­(3-ethylbenzothiazoline-6-sulfonic acid)
AI: atherogenicity index
TI: thrombogenicity index
